# Supplementary material for: Composition of time in movement behaviors and weight change in Latinx, Black and white participants
Source: PLoS One. 2021 Jan 8;16(1):e0244566. doi: 10.1371/journal.pone.0244566 (PMC7793306; doi:10.1371/journal.pone.0244566)
Supplement: S1 Table — (DOCX) [file pone.0244566.s002.docx]

**Supplemental Table 1.** Baseline characteristics of included compared to excluded participants

|  | Included (n=549) | Excluded (n=201) |
| --- | --- | --- |
| Characteristics |  |  |
|  | Arithmetic Mean (SD) | |
| Age (years) | 57 (9) | 57 (10) |
| Baseline weight (lbs.) | 179 (31) | 184 (39) |
|  | N (%) | |
| Women | 318 (57.9) | 123 (61.1) |
| White/Non-Latinx | 365 (66.6) | 167 (83.1) |
| Current smoker | 11 (2.0) | 4 (1.9) |
| Baseline body mass index |  |  |
| Underweight | 5 (0.9) | 2 (1.0) |
| Normal | 213 (38.8) | 75 (37.3) |
| Overweight | 199 (36.2) | 56 (27.9) |
| Obese | 132 (24.0) | 68 (33.8) |
