# Supplementary material for: Composition of time in movement behaviors and weight change in Latinx, Black and white participants
Source: PLoS One. 2021 Jan 8;16(1):e0244566. doi: 10.1371/journal.pone.0244566 (PMC7793306; doi:10.1371/journal.pone.0244566)
Supplement: S2 Table — (DOCX) [file pone.0244566.s003.docx]

**Supplemental Table 2.** Variation matrix of movement behaviors by sex

|  | Women | | | | |
| --- | --- | --- | --- | --- | --- |
| Movement behaviors | 1 | 2 | 3 | 4 | 5 |
| 1. Sedentary time | 0.000 | 0.206 | 0.243 | 1.468 | 0.055 |
| 2. Light PA |  | 0.000 | 0.235 | 1.441 | 0.111 |
| 3. Moderate PA |  |  | 0.000 | 1.093 | 0.196 |
| 4. Vigorous PA |  |  |  | 0.000 | 1.485 |
| 5. Sleep |  |  |  |  | 0.000 |
| Geometric mean of time spent in each behavior (min./day) | 583 | 277 | 55 | 8 | 518 |

|  | Men | | | | |
| --- | --- | --- | --- | --- | --- |
| Movement behaviors | 1 | 2 | 3 | 4 | 5 |
| 1. Sedentary time | 0.000 | 0.200 | 0.235 | 0.979 | 0.063 |
| 2. Light PA |  | 0.000 | 0.178 | 0.866 | 0.114 |
| 3. Moderate PA |  |  | 0.000 | 0.704 | 0.184 |
| 4. Vigorous PA |  |  |  | 0.000 | 0.954 |
| 5. Sleep |  |  |  |  | 0.000 |
| Geometric mean of time spent in each behavior (min./day) | 619 | 250 | 59 | 11 | 501 |

Variation matrix of all time-use behavior log-ratio variances. Note: A log-ratio variance close to 0 implies that time spent in the two behaviors are nearly proportional, hence, there is a high level of co-dependence between them.
