# Supplementary material for: Composition of time in movement behaviors and weight change in Latinx, Black and white participants
Source: PLoS One. 2021 Jan 8;16(1):e0244566. doi: 10.1371/journal.pone.0244566 (PMC7793306; doi:10.1371/journal.pone.0244566)
Supplement: S3 Table — (DOCX) [file pone.0244566.s004.docx]

**Supplemental Table 3.** Variation matrix of movement behaviors by race/ethnicity

|  | Latinx | | | | |
| --- | --- | --- | --- | --- | --- |
| Movement behaviors | 1 | 2 | 3 | 4 | 5 |
| 1. Sedentary time | 0.000 | 0.239 | 0.190 | 1.004 | 0.061 |
| 2. Light PA |  | 0.000 | 0.218 | 1.206 | 0.127 |
| 3. Moderate PA |  |  | 0.000 | 0.783 | 0.161 |
| 4. Vigorous PA |  |  |  | 0.000 | 1.085 |
| 5. Sleep |  |  |  |  | 0.000 |
| Geometric mean of time spent in each behavior (min./day) | 579 | 286 | 55 | 9 | 512 |

|  | Black (non-Latinx) | | | | |
| --- | --- | --- | --- | --- | --- |
| Movement behaviors | 1 | 2 | 3 | 4 | 5 |
| 1. Sedentary time | 0.000 | 0.200 | 0.281 | 2.125 | 0.072 |
| 2. Light PA |  | 0.000 | 0.303 | 2.263 | 0.135 |
| 3. Moderate PA |  |  | 0.000 | 1.561 | 0.287 |
| 4. Vigorous PA |  |  |  | 0.000 | 2.301 |
| 5. Sleep |  |  |  |  | 0.000 |
| Geometric mean of time spent in each behavior (min./day) | 625 | 255 | 50 | 6 | 504 |

|  | White (non-Latinx) | | | | |
| --- | --- | --- | --- | --- | --- |
| Movement behaviors | 1 | 2 | 3 | 4 | 5 |
| 1. Sedentary time | 0.000 | 0.202 | 0.229 | 1.060 | 0.056 |
| 2. Light PA |  | 0.000 | 0.187 | 0.946 | 0.103 |
| 3. Moderate PA |  |  | 0.000 | 0.804 | 0.169 |
| 4. Vigorous PA |  |  |  | 0.000 | 1.031 |
| 5. Sleep |  |  |  |  | 0.000 |
| Geometric mean of time spent in each behavior (min./day) | 595 | 264 | 59 | 10 | 512 |

Variation matrix of all time-use behavior log-ratio variances. Note: A log-ratio variance close to 0 implies that time spent in the two behaviors are nearly proportional, hence, there is a high level of co-dependence between them.
