# Supplementary material for: Composition of time in movement behaviors and weight change in Latinx, Black and white participants
Source: PLoS One. 2021 Jan 8;16(1):e0244566. doi: 10.1371/journal.pone.0244566 (PMC7793306; doi:10.1371/journal.pone.0244566)
Supplement: S4 Table — (DOCX) [file pone.0244566.s005.docx]

**Supplemental Table 4**. The association between activity composition (expressed as isometric log ratios) and percent weight change by sex and race/ethnicity

| Sex | Sum sq. | F | *p* |
| --- | --- | --- | --- |
| Women | **863** | **3.222** | **0.013** |
| Men | 70 | 0.646 | 0.630 |
| Race/Ethnicity |  |  |  |
| Latinx | **379** | **2.819** | **0.031** |
| Black | 23 | 0.156 | 0.960 |
| White | **941** | **4.152** | **0.003** |
| Joint* |  |  |  |
| White women | **1193** | **3.824** | **0.005** |
| White men | 212 | 2.087 | 0.085 |

Results from Wald chi square type II test of linear models. All models are adjusted for age, race/ethnicity or sex, average kcal/day (FFQ estimate), comorbidity score, and height. Results in bold are significant at 0.05. *Insufficient sample size for joint analyses among Latinx or Black participants.
