# Supplementary material for: Composition of time in movement behaviors and weight change in Latinx, Black and white participants
Source: PLoS One. 2021 Jan 8;16(1):e0244566. doi: 10.1371/journal.pone.0244566 (PMC7793306; doi:10.1371/journal.pone.0244566)
Supplement: S5 Table — (DOCX) [file pone.0244566.s006.docx]

**Supplemental Table 5**. The association between activity composition (expressed as isometric log ratios) and weight change by sex and race/ethnicity among those with BMI ≥25 kg/m^2^ at baseline

| Sex | Sum sq. | F | *p* |
| --- | --- | --- | --- |
| Women | **3446** | **3.424** | **0.010** |
| Men | 1012 | 1.838 | 0.125 |
| Race/Ethnicity |  |  |  |
| Latinx | 1062 | 2.543 | 0.055 |
| Black | 716 | 1.37 | 0.253 |
| White | **4985** | **5.177** | **<0.001** |
| Joint* |  |  |  |
| White women | **4351** | **3.329** | **0.013** |
| White men | **2221** | **3.878** | **0.006** |

Results from Wald chi square type II test of linear models. All models are adjusted for age, race/ethnicity or sex, average kcal/day (FFQ estimate), comorbidity score, and height. Results in bold are significant at 0.05. *Insufficient sample size for joint analyses among Latinx or Black participants.
